# Optical Coherence Tomography Characteristics of Quiescent Type 1 Neovascularization in Eyes with Nonexudative Age-related Macular Degeneration

**DOI:** 10.4274/tjo.galenos.2018.58908

**Published:** 2019-04-30

**Authors:** Jale Menteş, Şeyda Yıldırım

**Affiliations:** 1 Ege University Faculty of Medicine, Department of Ophthalmology, İzmir, Turkey; 2 Adıyaman University Training and Research Hospital, Department of Ophthalmology, Adıyaman, Turkey

**Keywords:** Age-related macular degeneration, quiescent type 1 choroidal neovascularization, spectral domain optical coherence tomography, en face optical coherence tomography

## Abstract

**Objectives:**

To describe the lesion characteristics of nonexudative, quiescent, asymptomatic type 1 neovascularization (NV) on B-scan and en face spectral domain optical coherence tomography (SD-OCT) in eyes with nonexudative age-related macular degeneration (AMD).

**Materials and Methods:**

In this retrospective, observational, consecutive case series, 27 patients who were already being followed and treated for exudative AMD in one eye were included in the study for their fellow eyes, which were initially nonexudative but developed exudative findings during follow-up. Initial B-scan and en face SD-OCT, fluorescein angiography (FA), and indocyanine green angiography (ICGA) images of these 27 eyes were examined retrospectively. The characteristic B-scan SD-OCT features of type 1 NV in this silent and asymptomatic stage were described.

**Results:**

The 27 eyes of 27 patients (13 males and 14 females; mean age 69.5±8.2 years) with nonexudative AMD had a mean best corrected visual acuity (BCVA) of 0.6±0.3 Snellen. Initial B-scan OCT images of all eyes (100%) showed retinal pigment epithelium (RPE) elevations and irregularities caused by a moderately reflective material in the sub-RPE space without fluid accumulation in the intraretinal/subretinal or sub-RPE space. Twenty-four eyes (88.8%) showed sub-RPE hyperreflective lesions consistent with type 1 NV on en face OCT images. While none of the eyes showed signs of type 1 NV in FA, macular plaque was observed in 8 eyes (29.6%) in ICGA. The mean time to onset of exudative findings was 8.3±4.03 months.

**Conclusion:**

In eyes with nonexudative AMD, there may be quiescent and asymptomatic type 1 NV lesions which do not yet show exudative changes. This NV has characteristic features on B-scan SD-OCT and can also be detected with en face OCT. Detection and close monitoring of these quiescent and inactive type 1 NV lesions during the asymptomatic, pre-exudative period are important for early treatment.

## Introduction

Neovascular age-related macular degeneration (NVAMD) is a destructive disease characterized by neovascularization (NV) in the macula that causes exudative changes affecting all retinal layers. It is currently among the leading causes of permanent and severe vision loss in people over 55 years old.^1,2,3^

In eyes with AMD, findings of new hemorrhages on clinical examination, subretinal, intraretinal, or sub-retinal pigment epithelium (RPE) fluid on spectral domain optical coherence tomography (SD-OCT) imaging and leakage consistent with these findings on fluorescein angiography (FA) are almost always indicative of active NV and are therefore accepted as signs of NVAMD.^1,2,3,4,5^

Neovascularization is classified into three types: Type 1 and type 2 NV originate from the choroidal circulation. Type 1 NV is located under the RPE, while type 2 NV occurs between the retina and RPE. Type 3 NV originates from the retinal circulation and is also called retinal angiomatous proliferation.^3^ Using multimodal imaging methods, it was shown that eyes with nonexudative AMD may have quiescent type 1 NV that does not yet show signs of activation (i.e., exudative symptoms).^2,3,4,5,6,7,8^ Knowing the characteristic SD-OCT features of these quiescent, subclinical type 1 NV lesions will facilitate early diagnosis and close follow-up of the lesions, enabling early treatment before severe losses in visual acuity.

SD-OCT is a noninvasive, fast, and easy to apply imaging method extensively used in ophthalmology clinics. It is repeated at almost every visit in patients being followed up and treated for NVAMD, and SD-OCT imaging plays a particularly essential role in making re-treatment decisions.

In this retrospective, observational clinical study, we examined B-scan and en face SD-OCT characteristic features during the quiescent, asymptomatic stage of type 1 NV lesions that later showed activation in patients who were under treatment and follow-up for NVAMD in one eye and developed exudative findings in their nonexudative AMD fellow eye.

## Materials and Methods

This retrospective, observational clinical case series included 27 eyes with nonexudative AMD of 27 patients who were being followed and treated for NVAMD in the fellow eye in the Ege University Faculty of Medicine Retina Unit between April 2013 and May 2016. The study was conducted using anonymized data and was approved by the Ege University Rectorate, Faculty of Medicine Dean’s Office, and Clinical Research Ethics Committee (18-2/39).

At initial presentation, patients diagnosed with NVAMD in one eye underwent best corrected visual acuity (BCVA) assessment using Snellen chart, intraocular pressure measurement, and biomicroscopic examinations of the anterior and posterior segments. In addition, color fundus photography, infrared fundus photography, fundus autofluorescence, B-scan SD-OCT (Topcon OCT-2000, Topcon, Tokyo, Japan), FA, and indocyanine angiography (ICGA) (Heidelberg Spectralis HRA+OCT, Heidelberg, Germany) were performed.

The patients received intravitreal anti-VEGF therapy in their eyes with NVAMD, and BCVA and SD-OCT assessment were repeated in both eyes at each follow-up visit. We retrospectively reviewed the initial B-scan and en face SD-OCT, FA, and ICGA images of 27 nonexudative eyes that developed exudative findings and active type 1 NV while the patients were receiving anti-VEGF therapy for NVAMD in the fellow eye. All SD-OCT images taken at presentation and during follow-up were analyzed to investigate signs of quiescent type 1 NV during the preclinical stage and identify common lesion characteristics.

## Results

The mean age of the 27 patients (13 males, 14 females) included in the study was 69.5±8.2 (55-87) years. Mean BCVA of the 27 eyes with nonexudative AMD was 0.6±0.3 (0.3-1) Snellen at first examination and remained stable until the emergence of exudative findings. The mean time between first examination and appearance of exudative signs was 8.3±4.03 (2-20) months.

**Figure 1:**
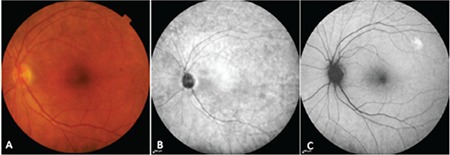
An eye with nonexudative age-related macular degeneration: A) Color fundus photograph shows pigment changes with no drusen; B) Infrared photography, C) Fundus autofluorescence image

At initial examination, both posterior segment slit-lamp biomicroscopic examination and color fundus and infrared fundus images demonstrated pigmentary changes in nearly all of 27 nonexudative AMD eyes, while no drusen were detected (Figure 1).

**Figure 2:**
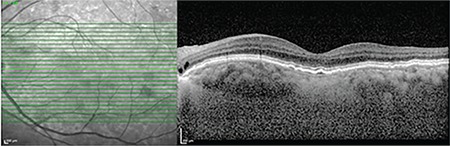
B-scan spectral domain optical coherence tomography shows elevations and irregularities in the retinal pigment epithelium layer, hyperreflective Bruch’s membrane, moderately reflective material between the two layers (double-layer sign), quiescent type 1 neovascularization

**Figure 3:**
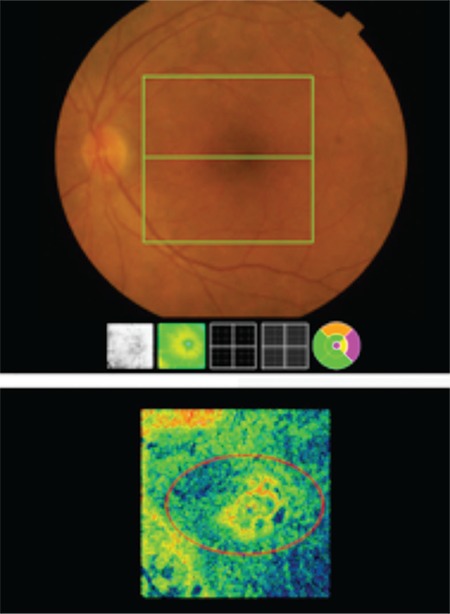
En face spectral domain optical coherence tomography shows hyperreflective lesion in the macula (red circle)

In B-scan SD-OCT imaging, features common to all eyes (100%) included a moderately reflective material between the RPE and Bruch’s membrane (BM) associated with slightly elevated, irregular, and undulating RPE (double-layer sign) and hyperreflective BM, but without SR, IR, or sub-RPE fluid accumulation (Figure 2). In en face SD-OCT (C-scan) imaging, 24 eyes (88.8%) showed sub-RPE hyperreflective lesions likely corresponding to type 1 NV (Figure 3).

**Figure 4:**
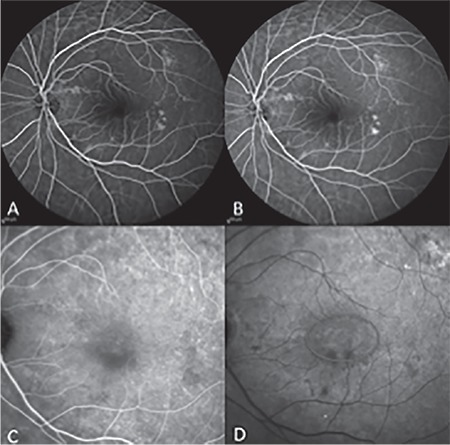
A and B) Early and late phase fluorescein angiography, no leakage. C and D) Early and late phase indocyanine green angiography, macular plaque evident in late phase (red circle)

**Figure 5:**
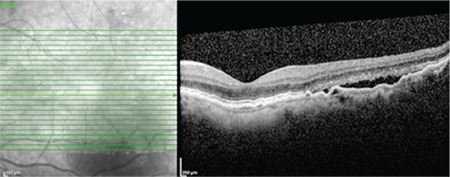
B-scan spectral domain optical coherence tomography shows subretinal fluid accumulation and active type 1 neovascularization 3.5 months after the initial diagnosis

In FA imaging, none of the eyes showed staining specific to macular type 1 NV or leakage until the late phase, but some eyes had focal hyperfluorescence limited to the macula. In ICGA imaging, mildly hypercyanotic macular plaques were detected in 8 eyes (29.6%) in the middle and late phases. The location of these plaques was consistent with the hyperreflective lesions detected in en face SD-OCT (Figure 4). One eye showed transformation to NVAMD with reduced BCVA, subretinal fluid accumulation, and emergence of exudative findings in SD-OCT at 3.5 months after the initial examination (Figure 5).

**Figure 6:**
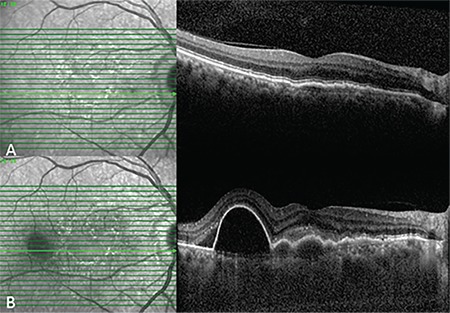
A) B-scan spectral domain optical coherence tomography shows moderately reflective material between the retinal pigment epithelium layer and hyperreflective Bruch’s membrane (double-layer sign) and quiescent type 1 neovascularization. B) B-scan spectral domain optical coherence tomography shows active type 1 neovascularization 9 months later

In another eye, type I NV that was quiescent in initial examination transformed into active type 1 NV after 9 months, and its B-scan SD-OCT features are shown in Figure 6.

## Discussion

In this study, using B-scan SD-OCT images we retrospectively determined that quiescent type 1 NV was present prior to the emergence of exudative symptoms in 27 nonexudative AMD eyes of 27 patients being followed and treated for NVAMD in the fellow eye, and we identified characteristic features of these subclinical lesions (100%). Some eyes exhibited findings associated with inactive NV on en face SD-OCT and ICGA imaging (88.8% and 29.6%, respectively), whereas no findings specific to the lesions were observed in FA imaging.

The most common type of NV in eyes with AMD is known to be type 1 NV, located in the sub-RPE space. This form originates in the choriocapillaris, penetrates BM over time, and expands between the RPE and BM, progressively growing and eventually causing fibrovascular pigment epithelial detachment.^2,3,4,8^ In our study, we found that these lesions show moderate reflectivity and cause the RPE to appear slightly elevated, irregular and undulating, leading to separation of the RPE and BM layers to create the “double-layer sign”. Various clinical studies using SD-OCT, ICGA, and optical coherence tomography angiography (OCTA) have shown that quiescent NV lesions not yet causing exudative findings may be present in eyes with nonexudative AMD, polipoidal choroidal vasculopathy, and angioid streaks.^2,3,4,5,6,7,8,9^

Hanutsaha et al.^10^ reported that of 432 patients who underwent FA and ICGA due to NVAMD findings in one eye, focal spot and macular plaque were detected in 11% of fellow eyes that exhibited only drusen. Exudative changes emerging in 24% of those eyes during the mean follow-up time of 21.7 months and the risk of progression to exudative disease was 2.60 times higher compared to eyes without plaques. Querques et al.^2^ presented the outcomes of 6-month follow-up with multimodal morphological and functional results obtained by SD-OCT, FA, ICGA, and microperimetry in 11 consecutive eyes with asymptomatic, quiescent choroidal NV. They found that NV lesions associated with slight RPE elevation and irregularity in SD-OCT but no fluid leakage exhibited a speckled pattern in the late phases of FA without leakage and appeared as plaques that uptake dye in middle/late phases of ICGA. In addition, they observed horizontal enlargement of the NV lesions during follow-up. Menteş et al.^7^ defined the multimodal imaging characteristics of quiescent type 1 NV lesions before the appearance of exudative findings in an eye with angioid streaks using SD-OCT, FA, ICGA, and OCTA.

Motulsky et al.^4^ referred to the appearance of RPE and BM as two hyperreflective bands with a small space between them on SD-OCT as the “double-layer sign.” They emphasized that the top layer is RPE, the bottom layer is BM, and the space between them is type 1 choroidal NV that is in a quiescent phase. The authors stated that in addition to SD-OCT, OCTA may be useful in detecting these lesions. Palejwala et al.^6^ detected quiescent choroidal NV using OCTA in 2 (6.25%) of 32 eyes with drusen and pigmentary changes in patients with NVAMD in the fellow eye. Roisman et al.^3^ investigated the presence of inactive NV in asymptomatic eyes using B-scan SD-OCT, FA, ICGA and swept-source (SS)-OCTA in 11 consecutive patients with nvAMD in one eye and asymptomatic, “intermediate AMD” in the fellow eye. In 3 (27.2%) of the 11 asymptomatic eyes with no signs of exudation on SD-OCT, they observed irregular RPE elevations on B-scan SD-OCT, macular plaque on ICGA, and the appearance of NV on SS-OCTA. The authors also concluded that a new classification delineating NVAMD and nonexudative AMD is needed. Similarly, de Oliveira Dias et al.^8^ investigated the presence of subclinical macular NV using SS-OCTA in 160 consecutive patients with NVAMD in one eye and nonexudative AMD in the fellow eye. They detected type 1 and 3 subclinical NV in 14.4% of the eyes, and found that all type 1 NV lesions caused RPE elevation in SS-OCT. In addition, they reported that although OCTA revealed no signs of sublinical NV, 3 eyes with RPE elevations in SS-OCT developed exudation 8 weeks later.

OCTA is a relatively new but extremely useful imaging modality that is noninvasive, reproducible, and detects NV based on blood flow.^3,4,5,6,8^ However, this method is still a developing technology and is not yet in widespread clinical use. Moreover, our study shows that B-scan SD-OCT imaging, one of the indispensable tools in retina clinics, is a reliable method that provides early and specific evidence of preclinical type 1 NV under the RPE even in eyes not yet exhibiting exudative symptoms.

## Conclusion

Based on the literature and the results of our study, we conclude that B-scan OCT is an easily applicable, reproducible, and sensitive imaging method that has positive specific findings in almost all cases for the early detection of quiescent (inactive) type 1 NV formations.
